# Dr. Heinrich Stern

**Published:** 1918-04

**Authors:** 


**Dr. Heinrich Stern,** St. Louis College of Physicians and Surgeons, 1899, died in New York City, Jan. 30, age 49. He was the editor of Archives of Diagnosis and Secretary of the American College of Physicians.

